# Anti-Inflammatory Effects of Natural Products on Cerebral Ischemia

**DOI:** 10.3389/fphar.2022.914630

**Published:** 2022-06-20

**Authors:** Yuanhong Shang, Zhe Zhang, Jinfeng Tian, Xiaokai Li

**Affiliations:** College of Biological and Chemical Engineering, Panzhihua University, Panzhihua, China

**Keywords:** anti-inflammatory effect, inflammatory mechanism, compound, cerebral ischemia, inflammation, natural product

## Abstract

Cerebral ischemia with high mortality and morbidity still requires the effectiveness of medical treatments. A growing number of investigations have shown strong links between inflammation and cerebral ischemia. Natural medicine’s treatment methods of cerebral ischemic illness have amassed a wealth of treatment experience and theoretical knowledge. This review summarized recent progress on the disease inflammatory pathways as well as 26 representative natural products that have been routinely utilized to treat cerebral ischemic injury. These natural products have exerted anti-inflammatory effects in cerebral ischemia based on their inflammatory mechanisms, including their inflammatory gene expression patterns and their related different cell types, and the roles of inflammatory mediators in ischemic injury. Overall, the combination of the potential therapeutic interventions of natural products with the inflammatory mechanisms will make them be applicable for cerebral ischemic patients in the future.

## Introduction

Stroke is a brain disease that causes numerous deaths and disabilities (Yuan et al., 2021), and it is the 3rd in disease mortality rate worldwide (Lo et al., 2003). Stroke is subdivided into ischemia and hemorrhage. Ischemic strokes account for approximately 87% of all deaths and were the focus of major drug trials (Rosenzweig et al., 2007). While ischemia is a pathologic condition in which blood flow is reduced to the point where normal cellular activity is disrupted (Adams et al., 2005; Sacco et al., 2006; Shang et al., 2013). The complex pathophysiology of cerebral ischemia primarily includes inflammatory pathways except ionic imbalances, neuroprotection, apoptosis, oxidative damage and angiogenesis (Deb et al., 2010; Dodd et al., 2021). There is growing evidences that some inflammatory mechanisms play a key role during the setting or acute phase of cerebral ischemia owing to subarachnoid hemorrhage, brain injury or cardiac arrest.

Inflammation, a dynamic tissue response mechanism to resist the invasion of pathogens, evolves over the course of evolution (Headland and Norling, 2015). The role of inflammatory mechanisms is crucial in stroke-related brain injury and tissue damage after ischemia (Przykaza, 2021). The understanding inflammatory of response mechanisms will help workers to choose appropriate intervention measures of blocking the cerebral ischemic injury cascade, and reducing neuronal death, and more efficiently preventing cerebral ischemia and treat disease in the theoretical and clinical applications. As a requirement of novel drug development for cerebral ischemia, there is a focus on the anti-inflammatory mechanisms and effects of natural products. Therefore, understanding and treatments of cerebral ischemic disease will be esential for scientists and clinicians to chooose the suitable anti-inflammatory measures and agents. Some specific natural products discovered from natural medical resources with the potentials in possessing anti-inflammatory effects on cerebral ischemia will increase the opportunity in laboratory and clinical trials.

## Methodology

Using exhaustive searches in the PubMed, China National Knowledge Internet (CNKI), Springer and Elsevier SDOL database, the present review gathered data of the research and trials from 2002 until February 2022 for 2 decades by adopting the following keywords and MeSH (medical subject heading) terms according to the Preferred Reporting Items for Systematic Reviews (Bayat et al., 2021). This review mainly summarized the inflammatory mechanisms and some representative natural products that have been commonly used in experimental research for the treatment of cerebral ischemia according to the inflammatory mechanism of ischemia.

## Inflammatory Mechanisms of Ischaemia

**FIGURE 1:**
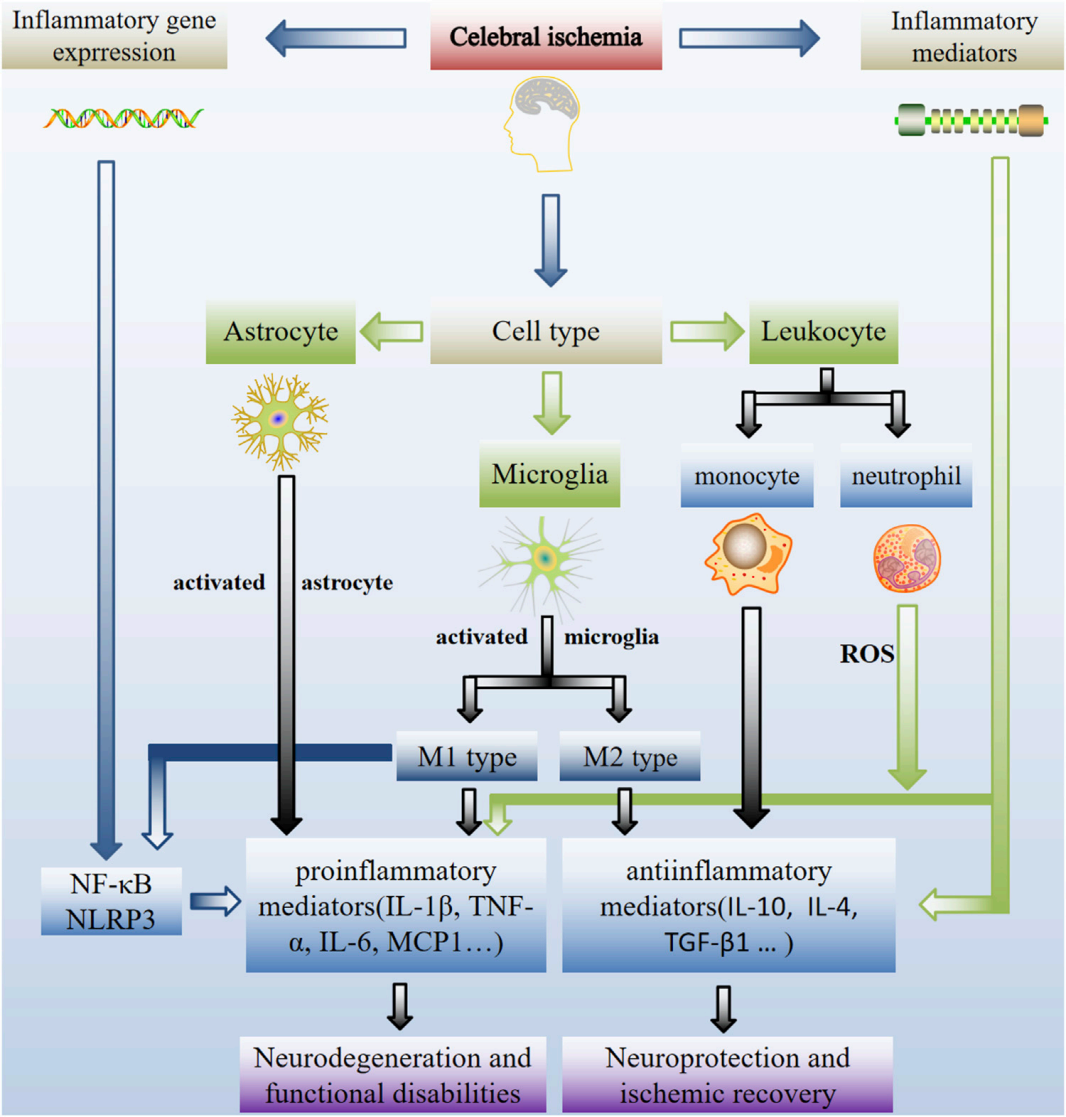
Schematic representation of inflammatory mechanisms in cerebral ischemia. Inflammation is crucial to the pathogenesis of ischemic stroke. Critical inflammatory and anti-inflammatory events involved in inflammatory gene expression, different cell types and inflammatory mediators contribute to ischemic injury. Inflammation inhibition is a possible treatment option for ischemic stroke-related neuro-inflammatory damage. Activation of local microglia and infiltrating macrophages from the compromised BBB occurs during cerebral ischemia. Microglia activation has two-way role (a double-edged sword). Stimulation the activity of microglia migrate to injured neurons and then switche to the M1 or M2 phenotype, which is implicated in nerve injury and repair. As a result, after an ischemic stroke, controlling the M1/m2 phenotype of microglia is critical for brain healing.

Inflammation acts as a critical role in the pathological progression of cerebral ischemia. Inflammation progresses in response to various stimuli are produced after ischemia. These progresses require activation and undergoes the rapid upregulation of multifarious genes, invasion of leukocyte (monocytes and neutrophils), and stimulation the activity of microglia, astrocytes, and endothelial cells following ischemia with the duration from hours to days (Huang et al., 2006; Zaleska et al., 2009), including many different inflammatory mediators and extracellular receptors. To avoid the evolution of acute resolving into persistent chronic inflammatory symptoms and further tissue damages, the inflammatory reaction should be actively resolved. Taken all together, the following discussions provided insights into the contributions of key inflammatory gene expression, different cell types, and inflammatory mediators to ischemic injury (Figure 1).

### Inflammatory Gene Expression

Ischemic injury in the brain parenchyma sets off an inflammatory cascade that exacerbates tissue damage. Each of these events may be affected by heredity, which makes it difficult to prove the classical pattern of heredity. Within minutes of occlusion, pro-inflammatory genes (heat shock proteins, transcription factors, adhesion molecules, chemokines and cytokines) that produce mediators of inflammation are upregulated (Raghay et al., 2020). Among these transcription factors, NF-κB (nuclear factor) regulated the expression of TNF-α (tumor necrosis factor α), NOS (nitric oxide synthase), IL-1β (interleukin-1β), IL-6 (interleukin-6), COX-2 (cyclooxygenase-2) and MCP1 (monocyte chemoattractant protein 1) *in vitro* (Deb et al., 2010). This has been demonstrated by reduced ischemic damage in mutant mice that targeted disruption of these genes (Adibhatla and Hatcher, 2008; Tănăsescu et al., 2008). Intercellular adhesion molecule-1 (ICAM-1) was found to appear in the postischemic no-reflow regions during cerebral ischemia. And it was also a mediator of leukocyte-endothelial cell adhesion (Sun et al., 2019). Matrix metalloproteinase (MMP) levels, especially MMP-9, have been found that associated with infarct growth, bleeding, transformation events, and neurological dysfunction (Montaner et al., 2003; Deb et al., 2010).

### Cell Types in Inflammation

The aggregation of inflammatory cells and mediators during cerebral ischemia is a characteristic of inflammation. After vascular occlusion, ischemic injury causes inflammatory cascade and further aggravates tissue injury (Amantea et al., 2014; Soliman et al., 2021). Reactive microglia, leukocytes (inflammatory cells) and macrophages have found to be recruited into the ischemic brain, and then resulting in inflammatory injury and the generation of inflammatory mediators by these cells, neurons and astrocytes.

### Microglia

Microglia play critical roles in brain inflammation following stroke, especially in the penumbral region of damage (Subedi and Gaire, 2021). Promoting the polarization of microglia and macrophages from the pro-inflammatory M1 to the anti-inflammatory M2 phenotype has been demonstrated to be a possible treatment for ischemic stroke (Li et al., 2021). M1 is generally considered to be neurotoxic, while M2 exhibits neuroprotective effects. Microglia are in a resting phase. And they account for 5–20% of glial cells (Deb et al., 2010). During ischemia, these cells get the significantly morphological and metabolic changes, as well as rapid and extensive genetic upregulations. These cells are the principal central nervous system (CNS) source of cytokines including transforming growth factor-β (TGF-β), TNF-α and IL-1β (Zarruk et al., 2018). Microglial cells have been indicated that can also produce pro-inflammatory cytokines and neuroprotective factors, such as TGF-β1, metallothionein-2 and erythropoietin. Because of the mixed nature of destructive and protective factors from microglial and astrocytes, the overall role of glia may differ at different time points following stroke insult, with protective or regenerative activities occurring days to weeks after the onset of ischemia. Several studies imply that activated microglia induce to injury (Wang et al., 2007). However, whether microglia cause damage following cerebral ischemia is not clear, and their anti-inflammatory potential is considered to be an significant neuroprotective factor after ischemic injury (Jin et al., 2017; Fukumoto et al., 2019; Gaire, 2021).

### Leukocytes

With increasing evidences, leukocytes are related to the pathogenesis of ischemic stroke. An increase in peripheral leukocyte counts are involved in the earliest inflammatory reactions in stroke. Neutrophils are the first leukocytes to respond, and their level is related to the infarct volume. From minutes to hours after reperfusion, neutrophil adhesion and migration have been observed in cerebral venules. After ischemia, the recruited cell population were switched from polymorphonuclear cells to mononuclear leukocytes and lymphocytes, and leukocyte recruitment continued days to weeks (Yilmaz et al., 2006; Gavins et al., 2007). Leukocyte-endothelial cell adhesion was prevented using adhesion molecule antibodies against stroke (Ishikawa et al., 2004). Therefore, leukocytes accumulate in the tissue after ischemia before tissue injury, and specific targeting of leukocytes provides substantive protection for ischemic injury.

### Astrocytes

In the human CNS, astrocytes are the main glial cell type and generally exceed the number of neurons. Astrocyte response is one of the earliest and the most significant changes in the CNS after ischemic injury. Astrocytes express different inflammatory mediators in inflammatory responses that may influence the outcome of ischemic injury (Falsig et al., 2006; Denes et al., 2008; Sofroniew, 2020).

### Inflammatory Mediators

Inflammatory mediators of cell adhesion to cerebral ischemia are primarily divided into cytokines and chemokines. Cytokines are a flock of small glycoproteins (∼25kd) from different sources. As a kind of cytokines, chemokines have chemotactic properties, which can stimulate cells to migrate to the source of chemokines. Chemokines polypeptides have been found to play the role in inflammation, immunological activation, cell differentiation, and cell death.

### Cytokines

Cytokines associated with inflammation in stroke, such as IL-1, TNF-α, IL-6, TGF-β and IL-10, are released and serve as intercellular messengers. Messengers mediate inflammatory and immunological responses. IL-6, IL-10 and TGF-β may have neuroprotective effect. IL-1 and TNF-α aggravate brain injury.

IL-1: IL-1 has two isoforms (IL-1α and IL-1β) and an endogenous inhibitor, IL-1 receptor antagonist (IL-1ra). Its pro-inflammatory properties were neutralized by IL-1ra administration (Emsley et al., 2005), and ischemic injury in IL-1ra deficient mice increased significantly (Pinteaux et al., 2006).

IL-10: an anti-inflammatory cytokine. Lower IL-10 levels are related to one of increased risks in stroke. Guizhi Fuling Capsule significantly upregulate the level of IL-10 mRNA and protein expression and its receptor in rats with focal cerebral ischemia and reperfusion (I/R) (Sun et al., 2015).

IL-6: a pro-inflammatory cytokine with unclear function in stroke. The clinical focus of IL-6 has revealed that IL-6 serum concentrations was an excellent independent predictor value for in-hospital mortality. (Rallidis et al., 2006).

TNF-α: TNF-α is a cellular signal protein involved in systemic inflammation and one of the cytokines involved in acute phase response. Activated macrophages primarily produce TNF-α. Its expression occurs peri-infarct areas in the ischemic core, adjacent distal areas in brain.

TGF-β: TGF-β may helpful for the rehabilitation therapy of ischemic stroke. Microglia secrete TGF-β1 that is reported to protect cultured neurons from ischemia-like injury (Lu et al., 2005). Its expression suggests a neuroprotective function in the penumbra during the recovery phase of cerebral injury.

### Chemokines

Chemokines play roles in cellular communication and inflammatory cell recruitment in host defense. Cytokines stimulate the production and release of chemokines C, CC, CXC, and CX3C [location based on the cysteine residues (C)] and MCP-1 (monocyte chemoattractant protein-1) in cerebral ischemia.

CX3C: CX3C expression was limited to surviving neurons around infarction and some endothelial cells during ischemia, and the CX3CR1 expression (CX3C receptor) was found only on microglia. Those suggest that fractalkine is involved in neuron-microglia signal transduction (Tarozzo et al., 2002).

MCP-1: MCP-1 overexpression significantly exacerbated the ischemic injury. This phenomenon is related to the activation of inflammatory cells. MCP-1 increased the cell permeability *in vitro*, and it generated changes in tight junction proteins, which suggest that it helps to ‘open’ the blood-brain barrier (BBB) (Stamatovic et al., 2005).

## The Anti-Inflammatory Effects of Natural Products

**FIGURE 2:**
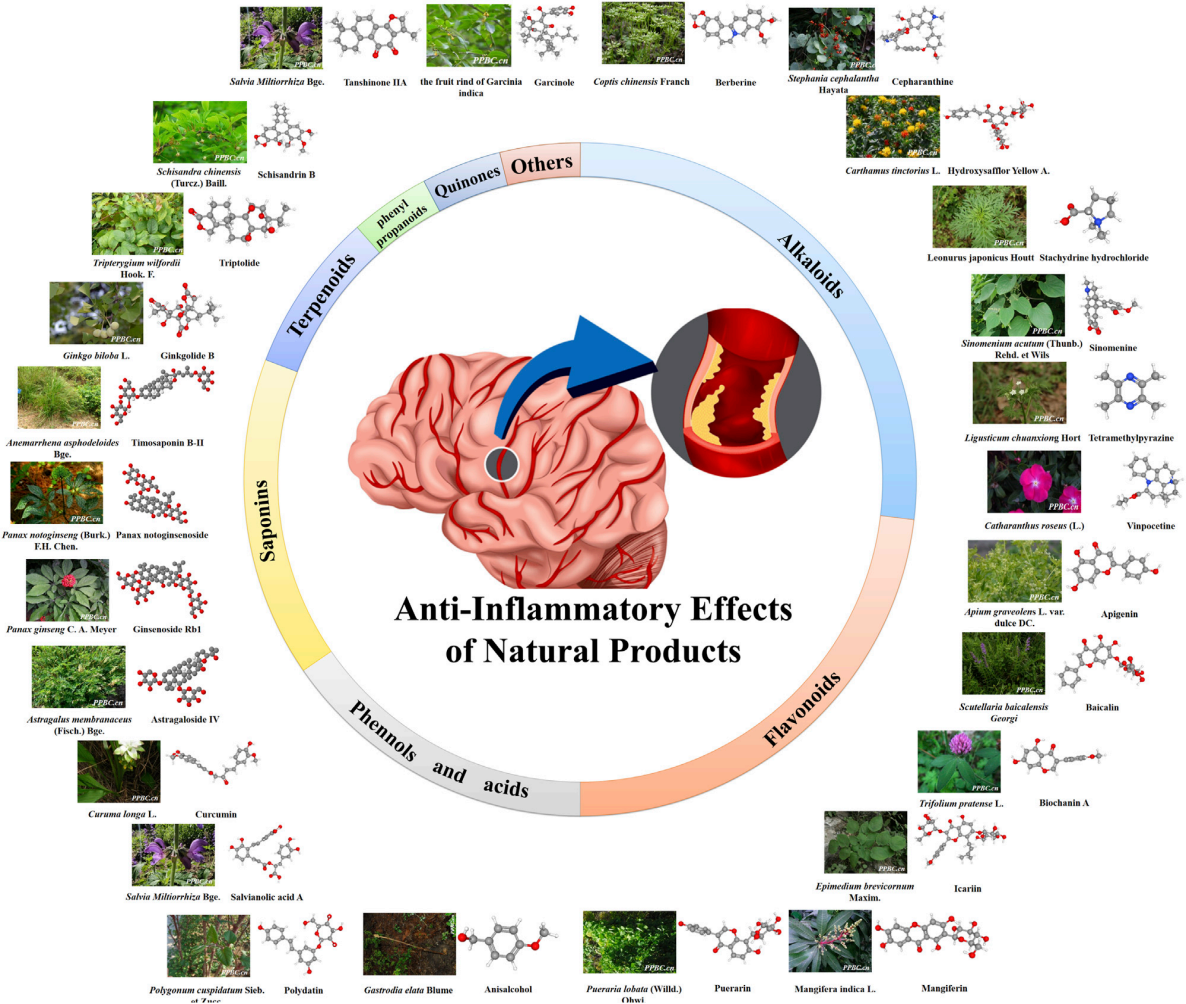
Anti-inflammatory effect of natural products. The color figures of medicine are viewed in www.iplant.cn. The club model comes from PubChem and Guidechem.

Historically, as folk medicines, natural products have been revealed the vast potentials for drug discoveries and the managements of various human diseases (Mu et al., 2020; Newman and Cragg, 2020; Atanasov et al., 2021). To date, the natural products used for anti-inflammation have been widely investigated and reported (Azab et al., 2016). These natural products with anti-inflammation effects are extensively distributed in different classes, e.g., flavonoids, terpenoids, saponins, phenylpropanoidss, anthraquinones, alkaloids, and phenols, etc., (Wang and Zeng, 2019) (Figure 2). These natural products exert significant anti-inflammatory effects *via* acting on different drug targets and cell signaling pathways.

### Anti-Inflammatory Effects of Flavonoids in Cerebral Ischemia

Flavonoids are low molecular weight phenolic compounds including A-, B- and C- rings. Most flavonoids that discovered so far have displayed a remarkable array of biochemical and pharmacological actions including anti-inflammatory effects (Romano et al., 2013). Several mechanisms have been proposed to explain the anti-inflammatory effects of flavonoids *in vivo*, including reducing the production of pro-inflammatory molecules and regulating the function of inflammatory cells (Serafini et al., 2010). The natural accuring flavonoids, such as apigenin, baicalin, biochanin A, icariin, mangiferin, and puerarin the flavonoid examples have exhibited anti-inflammatory effects of flavonoids (Table 1).

**TABLE 1:** Twenty-six active compounds from natural medicine with anti-inflammatory effects in cerebral ischemia.

Compound	Representative Sources	Cell Lines/Mode	Dose	Effects/Results	Safety evaluation (IC_50_/ID_50_/Others)	Reference
Anisalcohol	*Gastrodia elata* Blume	LPS-stimulated BV-2 cells	0.01–1 μM	TNF-α↓, JNK↓, IL-10↑, TGF-β↑	—	Xiang et al. (2018)
Apigenin	*Apium graveolens* L. var. dulce DC.	LPS-stimulated BV-2 cells	1, 5 and 10 μM	JNK↓, PGE_2_↓	12.5 μg/ml (MNTC)	Ha et al. (2008); Zhou et al. (2021)
Astragaloside IV	*Astragalus membranaceus* (Fisch.) Bge	(male ICR mice) bilateral common carotid artery occlusion/reperfusion (bCCAO/R)	10, 20 mg/kg	TNF-α↓, IL-1β↓, TLR4 ↓, NLRP3↑	1,000 μg/ml (MNTC, 90% vero cells)	Li M. et al. (2017); Indu et al. (2021)
Baicalin	*Scutellaria baicalensis Georgi*	(male SD rat) tMCAO LPS-induced RAW 264.7 cells	100 mg/kg 50, 100 and 200 μM	TNF-α↓, IL-6↓, p65↓, IL-10↑ IL-6↓, TNF-α↓, MCP1 (CCL2) ↓,IL-13↑, IL-1a↑	125 μg/ml (MNTC)	Xu et al. (2022); Zhou et al. (2021)
Berberine	*Coptis chinensis* Franch.(rhizomes of *Coptis chinensis*)	(male mice) tMCAO	25, 50 mg/kg	HMGB1↓, NF-κB↓, TLR4↓	IC_50_ values of berberine for U87 cells at 24, 48, and 72 h were 113.2, 62.15, and 33.07 mg/L, respectively	Zhu et al. (2018); Liu et al. (2020)
Biochanin A	*Trifolium pratense* L	(male SD rat) MCAO/R	10, 20 and 40 mg/kg	TNF-α↓, IL-1β↓, p38↑	IC_50_ value of 6.77 ±​ 0.83 μ​M in MGC-803 cells	Wang et al. (2015); Wang L. et al. (2020)
Cepharanthine	*Stephania cephalantha* Hayata	(male C57/BL6 mice) tMCAO OGD/R-treated BV-2 cells	10, 20 mg/kg 0.25–2.5 μM	NLRP3↓, IL-1β↓, IL-18↓	10 μ​g/ml (MNTC)	Zhao et al. (2020); Zhou et al. (2021)
Curcumin	*Curuma longa* L	(male Swiss albino mice) bCCAO/R	100 mg/kg	IL-6↓, NF-κB↓, MCP-1↓	IC_50_ values of curcumin for A431 cells were 19.2 μM for 24 h and 14.3 μM for 48 h	Hussein et al. (2020); Babaei et al. (2012)
Garcinol	fruit rind of Garcinia indica	(male SD rat) MCAO/R OGD/R-treated PC12 cells	5, 10 and 20 mg/kg 2.5, 5 and 10 μM	TNF-α↓, IL-1β↓ IL-6↓, TLR4↓, NF-κB ↓	The 40% garcinol (2000 mg/kg, ig) did not show any adverse effect at rats’ acute safety study	Kang et al. (2020); Majeed et al. (2018)
Ginkgolide B	*Ginkgo biloba* L	(male ICR mice) tMCAO/R	10, 20 and 40 mg/kg	TNF-α↓, IL-1b↓	—	Gu et al. (2012)
Ginsenoside Rb1	*Panax ginseng* C. A. Mey	(male SD rat)MCAO/R	12.5 mg/kg	TNF-α↓, IL-6↓, NF-κB ↓	—	Zhu et al. (2012)
Hydroxysafflor Yellow A	*Carthamus tinctorius* L	(male Wistar rats) MCAO/R	2, 4 and 8 mg/kg	NF-κB↓, ICAM-1↓	In the safe and well-tolerated of phase Ⅲ, it (75 mg/d) might be the optimal dose	Sun et al. (2010); Hu et al. (2020)
Icariin	*Epimedium brevicornum* Maxim	(male SD rat) MCAO/R	10, 30 mg/kg	IL-1β↓, NF-κB↓, TGF-β_1_↓, PPARα↑, PPARγ↑	3.0 ± 1.3 μM of IC_50_ in HEK293 cells	Xiong et al. (2016); Li et al. (2014)
Mangiferin	mango and papaya	(male Wistar rats) MCAO/R	25, 50 and 100 mg/kg	IL-10↑, L-1β↓, TNF-α↓	About 9 mM of IC_50_ in 3T3L1 cells	Zhang et al. (2016); Kumar et al. (2013)
Notoginsenoside R1	*Panax notoginseng* (Burk.) F.H.Chen	(male SD rat)the aorta clamped and rperfusion	30 mg/kg	IL-1β↓, IL-10↑, TNF-α↓	—	Ning et al. (2012)
Polydatin	*Polygonum Cuspidatum* Sieb. et Zucc	(male SD rat) pMCAO	50 mg/kg	NF-κB↓	IC_50_ at 24 and 48 h were 90 and 50 μmol/L in THP-1 cells	Ji et al. (2012); Wang et al. (2016)
Puerarin	*Pueraria lobata* (Willd.) Ohwi	(male Wistar rat) MCAO/R	18 mg/kg	NF-κB↓, ICAM-1↓	LD_50_ of oral puerarin is 2,000 mg/kg/d in rats	Lou et al. (2007); Chung et al. (2009)
Salvianolic acid A	*Salvia Miltiorrhiza* Bge	(male SD rat) MCAO/R	5, 10, and 20 mg/kg	NF-κB↓	48.4 ± 1.4 μM of IC_50_ on MMP-2 in Raw264.7 cells	Zhang et al. (2018); Zhang et al. (2014)
Schisandrin B	*Schisandra chinensis* (Turcz.) Baill	(male SD rat) MCAO/R	10, 30 mg/kg	TNF-α↓, IL-1β↓	—	Lee et al. (2012)
Stachydrine hydrochloride	*Leonurus japonicas* Houtt	(male KM mice) CCAO/R	15, 30 and 60 mg/kg	TNF-α↓, ICAM-1↓	—	Miao et al. (2017)
Sinomenine	*Sinomenium acutum* (Thunb.) Rehd. et Wils	(C57BL/6 mice) MCAO	10, 20 mg/kg	IL-1β↓, IL-6↓, IL-18↓, TNF-α↓	—	Qiu et al. (2016)
Tanshinone IIA	*Salvia Miltiorrhiza* Bge	OGD/R-treated BV-2 cells	0.5, 1 and 2 μM	NLRP3↓, IL-1β↓, IL-18↓	—	Cai et al. (2016)
Tetramethylpyrazine	*Ligusticum chuanxiong* Hort	(male SD rat) MCAO/R	20 mg/kg	PGE2↓	It (1,800 mg) is well tolerated in healthy volunteers of phase I	Liao et al. (2004); Zhao et al. (2021)
Timosaponin B-II	*Anemarrhena asphodeloides* Bge	(male SD rat) MCAO/R	100, 200 mg/kg	IL-10↑, IL-10R↑	No-observed-adverse-effect level is proposed to be 180 mg/kg (ig, rats)	Li et al. (2007); Lin et al. (2017)
Triptolide	*Tripterygium wilfordii* Hook. F	(male SD rat) intrahippocampal injection of LPS LPS-stimulated A172 cells	10–50 mg/kg 0.2–5 μM	COX-2↓, NF-_k_B↓	206.1 nM of IC_50_ in H295R cells	Dai et al. (2006); Xu et al. (2019)
Vinpocetine	*Catharanthus roseus* (L.)G. Don	(male Wistar rat) MCAO/R	10 mg/kg	NF-_k_B↓, TNF-α↓	—	Wang et al. (2014)

Note: "-" indicates that information on the potential toxicity and/or safety of the compound is not available or not in the literature that published in the last 20 years. MNTC, is a maximal non-toxic concentration of a compound that enables at least 80% of cells to survive.

### Anti-Inflammatory Effects of Alkaloids in Cerebral Ischemia

Alkaloids are nitrogen-containing organic compounds with an alkali like properties. They that are widely distributed in plants with diverse anti-inflammatory activities provide various potentials for the design and discovery of new anti-inflammatory drugs (Bai et al., 2021). Some alkaloids have strong anti-inflammatory activities and play important roles in the treatment of vascular disease (Alasvand et al., 2019). Anti-inflammatory alkaloids can be mainly divided into the following categories: isoquinoline alkaloids, indole alkaloids, pyridine alkaloids, terpenoid alkaloids, organic amine alkaloids, etc (Li S. et al., 2020). Several studies support the significance of the anti-inflammatory activity as an underlying mechanism for most of the pharmacological activities of the alkaloid (Tian et al., 2019; Geetha and Ramachandran, 2021). Alkaloids, such as berberine, cepharanthine, hydroxysafflor yellow A, sinomenine, tetramethylpyrazine, and vinpocetine, with anti-inflammatory effects in cerebral ischemia treatment, were summaried in Table 1.

### Anti-Inflammatory Effects of Saponins in Cerebral Ischemia

Saponins are glycosides that release sugar(s) and an aglycone (sapogenin) after acid hydrolysis. The aglycone of saponins can be triterpenoid or steroidal in nature. Steroidal saponins and saponins have been found to have diverse activities of inflammatory cytokines on a variety of inflammatory models (Passos et al., 2022). Studies support the anti-inflammatory activity as an underlying mechanism of the saponin in cerebral ischemia treatment (Sun et al., 2020; Yang F. et al., 2020; Xue et al., 2021). The anti-inflammatory sapnins, such as astragaloside IV, Ginsenoside Rb1, notoginsenoside R1, and Timosaponin B-II were summaried in Table 1.

### Anti-Inflammatory Effects of Terpenoids in Cerebral Ischemia

Terpenoids are secondary metabolites widely distributed in nature with diverse structures, and they represent a promising chemical group with potential beneficial effects in neurological diseases in view of the pleiotropic effects on cell death and survival, which consolidate their therapeutic value (Gonzalez-Cofrade et al., 2019; Proshkina et al., 2020). Several studies support the therapeutic potential compounds known to be effective among various inflammatory diseases (Downer, 2020; Khoshnazar et al., 2020; Kim et al., 2020). Terpenoids, combined with the studied mechanism and commonly used drugs, may be new strategy for further anti-inflammatory treatment. The anti-inflammatory effects of terpenoids including ginkgolide B and triptolide are reviewed in cerebral ischemic treatment (Table 1).

### Anti-Inflammatory Effects of Phenols and Acids in Cerebral Ischemia

(Poly) Phenols are natural substances with variable phenolic structures, and they are generally known to possess potent anti-inflammatory properties. It has been found that phenols can affect the polarization of M1/M2 *via* TLR-4/NF-κB pathway and exert anti-inflammatory properties to treat ischemic stroke (Li et al., 2022). The anti-inflammatory effects of phenols and acids including anisalcohol, curcumin, polydatin and salvianolic acid A are reviewed in cerebral ischemic treatment (Table 1).

### Anti-Inflammatory Effects of Quinones, Phenylpropanoids and Others in Cerebral Ischemia

In addition to the types of compounds mentioned above, phenylpropanoids, quinones and others are a large group of organic compounds in the nature (Table 1). Anti-inflammatory effects of them are found and reported (Gui et al., 2020; Yang et al., 2021).

## Major Anti-Inflammatory Natural Products From Natural Medicines

Natural products have been long used as folk drugs all over the world although the resources and applications vary among different regions. In China, many natural medicines have been accustomed to treat stroke for years. The direct experience gained from human subjects provides a huge resource for new drug candidates and treatment methods for stroke. Therefore, this review primarily discussed several representative natural products (compounds) with anti-inflammatory effects in cerebral ischemia treatment.

Anisalcohol is a phenolic compound that was originally isolated from *Gastrodia elata* Blume (Chen H. L. et al., 2020). Anisalcohol has ability to significantly decrease TNF-α and increase IL-10 and TGF-β in lipopolysaccharide (LPS)-stimulated BV-2 cells (a murine microglial cell line). Anisalcohol has also found to inhibit c-Jun N-terminal kinase (JNK) phosphorylation and suppress NF-κB activation. Therefore, anisalcohol reduces the generation of inflammatory mediators and cytokines by suppressing NF-κB and mitogen-activated protein kinase (MAPK) activation (Xiang et al., 2018).

Apigenin is one of the active ingredients in the leaves of *Apium graveolens* L. var. dulce DC. Apigenin decreased prostaglandin E_2_ (PGE_2_) levels by inhibiting COX-2 expression and mediating inflammation in LPS-stimulated BV-2 cells. They found that LPS induced p38 MAPK and apigenin inhibited the phosphorylation of JNK in BV-2 microglia, suggesting that it played an anti-inflammatory role through p38 MAPK and JNK. However, apigenin had no significant effect on extracellular signal-regulated kinase activation (Ha et al., 2008).

Astragaloside IV is an ingredient isolated from the dried roots of *Astragalus membranaceus* (Fisch.) Bge (Zhang et al., 2020). As shown by Morris water maze test, bilateral common carotid artery occlusion in mice can lead to serious memory impairment. Oral administration of astragaloside IV (10 and 20 mg/kg, once daily, beginning 7 days before surgery and continued for 7 days after surgery) could reduce Toll-like receptor-4 (TLR4) expression and its downstream adaptor proteins, including tumor necrosis factor receptor associated factor-6 (TRAF6), so NF-κB phosphorylation was inhibited as a result (Li M. et al., 2017). These findings show that astragaloside IV protects against transient cerebral I/R by suppressing the TLR4 signaling pathway, in part through its anti-inflammatory properties.

Baicalin is an important flavonoid that is extracted from the root of *Scutellaria baicalens*is Georgi (Chen H. et al., 2020; Li Y. et al., 2020). The report was found that baicalin inhibited the secretion of NO, IL-6, TNF-α, and CCL22 (C-C motif chemokine ligand 22) in macrophages, promoted the secretion of IL-13, IFNG (interferon gamma), and IL-1a *in vitro* (Xu et al., 2022). It inhibited CCL2 expression, reduced the phosphorylation levels of p65 and IκBα protein, and downregulated the level of CCR2 *in vivo*. Meanwhile, it discovered that baicalin had a neuroprotective effect, most likely via blocking NF-κB p65 activation, which improved neurological functioning and reduced the extent of cerebral infarction (Xue et al., 2010). The anti-inflammatory and anti-apoptotic benefits of baicalin were validated by Tu et al. (Tu et al., 2009), and the molecular mechanism may be related to the downregulated levels of iNOS, COX-2, and cleaved caspase-3 protein, as well as the enhanced enzymatic activity of myeloperoxidase (MPO). Current studies indicate that baicalin protects from ischemic injury by inhibiting of NF-κB–mediated inflammation.

Berberine is a non-basic and quaternary benzylisoquinoline alkaloid in berberine-containing plants (the berberidaceae family) worldwide, and it is an active ingredient isolated from the dried roots of *Coptidis rhizoma* (rhizomes of *Coptis chinensis*) in China (Neag et al., 2018). In I/R injury, berberine dramatically lowered the levels of pro-inflammatory cytokines, which was prevented by a SIRT1 inhibitor (Yu et al., 2016). It found that pretreatment with berberine dose-dependently inhibited high-mobility group box 1 (HMGB1) and NF-κB translocation from the nucleus to the cytoplasm in cerebral I/R injury and decreased Toll-like receptor 4 (TLR4) expression in ischemic cortical tissue (Zhu et al., 2018). According to new findings, berberine’s anti-inflammatory benefits against the injury are aided by reducing the activation of the HMGB1/TLR4/NF-κB signaling pathway. In clinical studies, berberine administration lowered serum levels of IL-6 and macrophage migration inhibitory factor, as well as plasma levels of C-reactive protein, TNF-α, and IL-6 (Li et al., 2016; Liu et al., 2019). Berberine appears to reduce I/R injury by reducing excessive inflammatory responses in patients according to recent research. Berberine could be employed as an alternate therapeutic technique for I/R injury management.

Biochanin A is an O-methylated natural isoflavonoid isolated from *Trifolium pratense* L. (red clover). In a rat model, the anti-inflammatory properties of biochanin A against I/R damage were examined. TNF-α and IL-1β expression, as well as p38 phosphorylation, were assessed using RT-PCR or WB. As a result, TNF-α and IL-1β levels were markedly elevated following I/R injury, and biochanin A therapy significantly reduced these inflammatory processes. Meawhile, the increase in p-p38 levels in I/R brain tissue was reduced by biochanin A (Wang et al., 2015). These results suggest that biochanin A protects against focal cerebral I/R in rats via inhibition of p38-mediated inflammatory responses.

Cepharanthine is a bibenzylisoquinoline alkaloid isolated from *Stephania cephalantha* Hayata (Huang H. et al., 2014). Cepharanthine reduced microglia activation in transient middle cerebral artery occlusion (tMCAO) mouse models and in hypoxia, glucose deprivation/reoxygenation microglia models (OGD/R). Cepharanthine reduced microglial activation. Following tMCAO, cepharanthine reduced elevation of NLR family pyrin domain containing 3 (NLRP3) immunoreactivity in Ibal-labeled microglia as well as total Iba1 and NLRP3 expression in the brain, according to immunofluorescence labeling. Cepharanthine reduced the overproduction of the M1 microglia-regulated pro-inflammatory cytokines IL-1 and IL-18 generated by tMCAO and OGD/R (Zhao et al., 2020). Cepharanthine reduced cerebral I/R injury by reducing microglial activation and inflammation generated by the NLRP3 inflammasome.

Curcumin is an effective ingredient from the dried roots of *Curuma longa* L. Curcumin (100 mg/kg) decreased the degree of neutrophilic granulocyte infiltration in cerebral tissues and inhibited TNF-α expression in a cerebral I/R injury model (Lei et al., 2010). Treatment with curcumin (IP) decreased IL-6, NF-κB and MCP-1 levels after I/R injury (Hussein et al., 2020). These findings suggest that curcumin has a neuroprotective role by inhibiting the inflammatory reaction.

Garcinol is a benzophenone compound isolated from the fruit rind of Garcinia indica (Balasubramanyam et al., 2004). In MCAO/R-induced animals and OGD/R-treated cells of models, garcinol reduced model-induced inflammation, including inhibiting the production of IL-6, IL-1β, and TNF-α, and it also inhibited TLR4 and NF-κB (p65) expression. The data indicate that garcinol protects against cerebral I/R injury. And garcinol attenuates inflammation by suppression of the TLR4/NF-ĸB signaling pathway (Kang et al., 2020).

Ginkgolide B is a compound extracted from the dried leaves of *Ginkgo biloba* L. The tMCAO model of mice was made by an intraluminal filament technique. Ginkgolide B (10, 20 and 40 mg/kg in 2 h after MCAO, iv) inhibited I/R-induced NF-κB, microglial activation and the production of pro-inflammatory cytokines (Gu et al., 2012). Taken together, the information indicates that ginkgolide B has an anti-inflammatory function by inhibition of NF-κB pathway.

Ginsenoside Rb1 (Rb1) is one of the primary active compounds of *Panax ginseng* C. A. Mey. Male Sprague-Dawley (SD) rats were given Rb1 (12.5 mg/kg/d) intranasally for 7 days before being subjected to transient blockage of the right middle cerebral artery and reperfusion. The rats were slaughtered 6 hours, 12 hours, 24 hours, and 72 hours following reperfusion, and brain tissues were taken for testing. Key inflammatory aspects of CNS, such as inflammatory cells, pro-inflammatory cytokines, and transcription factors, were used to assess Rb1’s neuroprotection. Rb1 inhibited microglia activity in the penumbra from 24 to 72 h after reperfusion, as well as microglia conversion to phagocytic microglia. In the Rb1 group, the mRNA and protein level of TNF-α was lower 12 h after reperfusion. From 6 to 72 h, Rb1 partially reduced NF-κB pathway activation (Zhu et al., 2012). These findings suggest that local inflammation suppression during cerebral ischemia may be one mechanism contributing to Rb1’s neuroprotective benefits.

Hydroxysafflor Yellow A is a component of flavonoids extracted from the flower of the safflower plant *Carthamus tinctorius* L. Hydroxysafflor Yellow A (2, 4, 8 mg/kg, respectively, iv) prevented brain injury in a focal cerebral I/R model by inhibiting thrombin generation. Hydroxysafflor yellow A treatment suppressed NF-κB p65 nuclear translation and p65 binding activity, as well as ICAM-1 mRNA and protein levels and neutrophil infiltration. Hydroxysafflor yellow A enhanced the number of CA1 pyramidal cells in the hippocampus CA1 and lowered plasma angiotensin II levels, all of which improved neurological deficit scores (Sun et al., 2010). Another study revealed the neuroprotective effect of hydroxysafflor yellow A on GSK-3β/NF-κB-mediated inflammatory pathways (Yang X. et al., 2020). These findings demonstrated that hydroxysafflor yellow A had an anti-inflammatory effect by reducing angiotensin II content or regulating NF-κB.

Icariin is a natural compound of flavonoids extracted from *Epimedium brevicornum* Maxim. Pretreatment with icariin (10, 30 mg/kg) decreased NF-κB activation, IL-1β and TGF-β_1_ protein levels in the cerebral I/R model of rats. Icarin also increased the levels of peroxisome proliferator-activated receptor (PPARα and PPARγ) protein in this study. These results suggest that icariin protects rats from ischemic stroke by inhibiting inflammatory responses mediated by NF-κB, PPARα and PPARγ (Xiong et al., 2016).

Mangiferin is a natural compund found in papaya and mango (the Anacardiaceae and Gentianaceae families). Mangiferin (25, 50, 100 mg/kg, respectively, iv) increased IL-10 levels and reduced IL-1β and TNF-α levels in the rat brain tissues with cerebral I/R injury. These results demonstrate that mangiferin has a significant protective ability that may be its effect to inhibit the overproduction of inflammatory cytokines (Zhang et al., 2016).

Notoginsenoside R1 is an active component extracted from the dried roots of *Panax notoginseng* (Burk.) F.H. Chen. After administration notoginsenoside R1 for 3 days, bilateral CCA were occluded with artery clip for 20 min followed by reperfusion for 24 h in C57BL/6 mice, notoginsenoside R1 reduced both TNF-α and ICAM-1 mRNA (Huang et al., 2015). Panax notoginsenoside (30 mg/kg, including notoginsenoside R1) was injected intraperitoneally approximately 30 min before aortic clamping and reperfusion, and it decreased the expression of IL-1β, IL-10, and TNF-α levels in this acute spinal cord I/R injury model, as shown by immunohistochemistry and Western blotting (Ning et al., 2012). These results indicated that panax notoginsenoside effectively decreased inflammation in damaged spinal cord tissues, but it did not completely ameliorate the symptoms.

Polydatin is a stilbene chemical isolated from *Polygonum cuspidatum* Sieb. et Zuccdried.'s roots (Ji et al., 2012; Tang and Tan, 2019). Polydatin (50 mg/kg) decreased the number of cells positive for NF-κB in an SD rat model of permanent MCAO (pMCAO) compared to the blank control group 24 and 72 h after pMCAO (Ji et al., 2012). These findings indicate that polydatin inhibits the inflammation in response to exert its neuroprotective effects after pMCAO.

Puerarin is one of the main active ingredients from the dried roots of *Pueraria lobata* (Willd.) Ohwi. (Wei et al., 2014). A cerebral I/R injury model was created using MCAO in rats. Puerarin (18 mg/kg) was administered just before occlusion and immediately after reperfusion, and it decreased ICAM-1 protein level and the nuclear translocation of the NF-κB p65 subunit (Lou et al., 2007). These effects may be due to its inhibition of the neutrophil-mediated inflammatory response.

Salvianolic acid A is one of the main active components isolated from *Salvia Miltiorrhiza* Bge. MCAO operation was used to create a focal cerebral I/R model for 1.5 h of ischemia, followed by reperfusion after 24 h. Salvianolic acid A (5, 10, and 20 mg/kg, respectively, i. v.) administration kept off cerebral NF-κB p65 activation and released the inflammatory response (Chien et al., 2016; Zhang et al., 2018; Yang et al., 2022). These findings suggest that salvianolic acid A attenuates I/R-induced rat brain injury by protecting the BBB via anti-inflammation.

Schisandrin B is a compound isolated from the dried fruits of *Schisandra chinensis* (Turcz.) Baill. Schisandrin B (10, 30 mg/kg, i. p.) was administered twice 30 min before the onset of ischemia and 2 h after reperfusion. Schisandrin B therapy suppressed the TNF-α and IL-1β protein expression in ischemic hemispheres (Lee et al., 2012). These findings show that schisandrin B therapy protects rats from neurodegeneration by suppressing inflammation.

Stachydrine hydrochloride is an alkaloid isolated from *Leonurus japonicus* Houtt. Stachydrine hydrochloride (15, 30 and 60 mg/kg) was administered in a repetitive cerebral ischemia reperfusion mouse model, and it reduced the levels of TNF-α, ICAM-1, and MPO. These results suggest that stachydrine hydrochloride inhibits inflammatory reactions after ischemia (Miao et al., 2017).

Sinomenine is an isoquinoline-type alkaloid originally extracted from the *Sinomenium acutum* (Thunb.) Rehd. et Wils and *S. acutum* (Thunb.) Rehd. et Wils var. *cinereum* Rehd. et Wils. (Zhao et al., 2015). Sinomenine inhibited pro-inflammatory factors including IL-1β, TNF-α, IL-6 and IL-18 to protect BBB integrity and improve neurological functions (Qiu et al., 2016). It reduced the activation of microglia and astrocytes, and it also decreased inflammasome-related molecules (NLRP3, ASC, and caspase-1) and proinflammatory cytokines in ischemic brains *in vivo* and cultured microglia *in vitro* (Yang et al., 2016). Therefore, sinomenine inhibits inflammatory reactions in ischemic stroke treatment.

Tanshinone IIA is an active ingredients isolated from *Salvia Miltiorrhiza* Bge. (Subedi and Gaire, 2021). Tanshinone IIA has an anti-inflammatory impact in N2a cells, where it increases cell viability and reduces inflammation via the PI3K/Akt/mTOR signaling pathway (Wang J. et al., 2020). Another study in OGD/R-exposed microglia found that Salvia Miltiorrhiza extract containing tanshinone IIA significantly reduced inflammatory responses, as shown by lower expression of NLRP3, caspase-1, IL-1, and IL-18 (Cai et al., 2016). Tanshinone IIA also had anti-inflammatory effects in OGD/R-activated astrocytes by reducing proliferation, GFAP staining, HIF-1 expression, and SDF-1, ERK, and Akt signaling (Huang X. et al., 2014). It enhanced cell viability while lowering NO production and NF-κB signaling (Dong et al., 2009). These studies suggest that tanshinone IIA prevents inflammatory responses after ischemia.

Tetramethylpyrazine is an active compound from *Ligusticum chuanxiong* Hort . The MCAO model was produced in rats by occluding the right middle cerebral artery for 90 min and then reperfusing for 3 days, using tetramethylpyrazine (20 mg/kg) administered intraperitoneally 60 min before the occlusion. Tetramethylpyrazine significantly reduced inflammatory cell activation and pro-inflammatory mediator synthesis in the brain after ischemia and reperfusion. In cultured glial cells, it reduced inflammation generated by lipopolysaccharide and interferon, as well as the formation of prostaglandin E2 (PGE2) (Liao et al., 2004). These findings show that one of tetramethylpyrazine’s neuroprotective properties to prevent brain injury is its anti-inflammatory capability.

Timosaponin B-II is an active ingredient isolated from the dried roots of *Anemarrhena asphodeloides* Bge. Timosaponin B-II treatment significantly increased IL-10 and IL-10R mRNA expression in an SD rat model of focal cerebral I/R produced by transient MCAO (2 h) (Li et al., 2007). The results demonstrated that the anti-dementia effect of timosaponin B-II is at least partially due to its anti-inflammatory properties.

Triptolide is a biologically active natural chemical derived from the *Tripterygium wilfordii* Hook plant. F. Triptolide (0.2–5 mg/L) suppressed the astroglial response and NF-κB/DNA binding activity *in vitro*, while triptolide (10–50 mg/kg) lowered LPS-induced COX-2 expression *in vivo* and *in vitro* (Dai et al., 2006). These findings imply that triptolide protects against neuroinflammation by suppressing COX-2 production, at least in part through suppression of the NF-κB signaling pathway.

Vinpocetine is one of the major active ingredients in *Catharanthus* roseus (L.) Don. Vinpocetine (10 mg/kg) was intraperitoneally injected in a murine model of transient MCAO (2 h) and then reperfusion. Vinpocetine decreased the expression of NF-κB and TNF-α at 24 h and 3 days and inhibited the inflammatory response after cerebral I/R (Jeon et al., 2010; Wang et al., 2014).

## Strcture-Activity Relationship Between Inflammation and Natural Products

Structure activity relationship (SAR) has been demonstrated as a useful tool for investigating the bioactivities of various classes of compounds (Li X. et al., 2017). The SAR of biological activity is mainly related to the type (or basic parent nucleus) of compounds and the position of main functional groups (Xie et al., 2021; Zhou et al., 2021). Flavonoids are based on 2-phenylchromone as the skeleton. The position of C-5 and 7 on the ring A of flavonoids that coupled with hydroxyl groups has found to affect the cell secretion process, mitosis and cell interactions with strong anti-inflammatory effects (Xie et al., 2021). Forthermore, C_5_-OH, C_7_-OH, C_2_ = C_3_ and C_4_ = O functional groups that present in the flavonoids has also been indicated to performa greater anti-inflammatory effect. C_3_-OH or glycosylation group at the ring A has greatly effection in the decrease the anti-inflammatory (Zhang et al., 2019). For alkaloids and other types of natural compounds mentioned above, due to the number or complex structure of compounds, this review did not draw a suitable SAR.

## Conclusion

Even though cerebral ischemia as a serious issue with global health concern, the lack of effective drug options still limit its treatment (Gaire, 2021). The present study provides a wealth of information to elucidate the important role of inflammation in ischemic brain injury. Natural medicines with anti-inflammatory effects in cerebral ischemia have great potential for treatment based on the above mentioned 26 active ingredients in experimental studies (Table 1). This review fully demonstrated that compounds of natural medicine have protective effects against cerebral ischemia *via* anti-inflammatory mechanisms.

Inflammation is a critical mediator of cerebral ischemic injury, and it is increasingly regarded as the important factor to the pathological processes in cerebral ischemia. Experimental anti-inflammatory strategies to diminish cerebral ischemic injury have certainly been useful. In-depth study of the inflammatory response of cerebral ischemic injury offers a novel method for the prevention and treatment. However, whether inflammation is harmful or useful may depend on the severity of the ischemia and the stage of ischemia in which inflammatory responses contribute, and detailed mechanisms of inflammation-induced injury or recovery are far from complete. In the laboratory, a lot of time and money has gone into studying the mechanisms, but it hasn't translated into effective treatments in the clinic. Compound preparation is difficult to prepare for the international market because internationality only admits the curative effect of monomers, which is worthy of attention in the future.

The use of natural medicine compounds to study the anti-inflammatory effect of cerebral ischemia should strengthen the recent research achievements of physiology, pathology, immunology, molecular biology, neurobiology, neuroscience and omics methods. Using the research method of Western-style drugs and recognizing each step of the inflammatory response leads to the possibility of developing a new compound from natural medicines that interferes with a specific inflammatory mechanism of ischemic injury and creating a new situation that prevents and treats cerebral ischemic injury using compounds of natural medicines for anti-inflammatory effects. Review of the inflammatory mechanism and anti-inflammatory effect of the above mentioned 26 compounds revealed great new progress and breakthroughs in the future, thanks to a strong scientific research capability to protect cerebral ischemia with natural products based on the inflammatory theory.
